# Association of Professional Football Cumulative Head Impact Index Scores With All-Cause Mortality Among National Football League Players

**DOI:** 10.1001/jamanetworkopen.2020.4442

**Published:** 2020-05-11

**Authors:** Brittany L. Kmush, Madeline Mackowski, Justin Ehrlich, Bhavneet Walia, Arthur Owora, Shane Sanders

**Affiliations:** 1 Department of Public Health, Syracuse University, Syracuse, New York; 2 Department of Sport Analytics, Syracuse University, Syracuse, New York; 3 Currently with Department of Epidemiology and Biostatistics, Indiana University Bloomington, Bloomington

## Abstract

**Importance:**

Long-term adverse health outcomes, particularly those associated with repetitive head impacts, are of growing concern among US-style football players in the US and Canada.

**Objective:**

To assess whether exposure to repetitive head impacts during a professional football career is associated with an increase in the risk of all-cause mortality.

**Design, Setting, and Participants:**

This retrospective cohort study included 13 912 players in the 1969 to 2017 National Football League (NFL) seasons. All cause-mortality up until July 1, 2018, was included. Data collection was performed from July 13, 2017, to July 1, 2018, as reported in the Pro Football Reference.

**Exposures:**

The main exposure was a professional football cumulative head impact index (pfCHII). The pfCHII was measured by combining cumulative padded practice time and games played summed during seasons of play reported in the Pro Football Reference and a player position risk adjustment from helmet accelerometer studies.

**Main Outcomes and Measures:**

Demographic characteristics except for the pfCHII were calculated for 14 366 players with complete follow-up. The pfCHII was calculated for 13 912 players (eliminating the 454 specials teams players). Cox proportional hazards regression was used to compare hazard ratios (HRs) of death by repetitive head impacts. Analyses were unadjusted and adjusted for birth year, body mass index, and height.

**Results:**

Among 14 366 NFL players who had follow-up for analysis, the mean (SD) age was 47.3 (14.8) years, the mean (SD) body mass index was 29.6 (3.9), and 763 of 14 366 players (5.3%) had died. Among 13 912 players in the pfCHII analysis, the median pfCHII was 32.63 (interquartile range, 13.71-66.12). A 1-log increase in pfCHII was significantly associated with an increased hazard of death for the 1969 to 2017 seasons (HR, 2.02; 95% CI, 1.21-3.37; *P* = .01) after adjustment. The quadratic pfCHII was also statistically significant (HR, 0.91; 95% CI, 0.85-0.98; *P* = .01), indicating that the hazard of death increased at a decreasing rate, whereas the pfCHII increased.

**Conclusions and Relevance:**

The findings suggest that an increase in repetitive head impacts is associated with an increased hazard of death among NFL players. Reduction in repetitive head impacts from playing football or other activities through additional rule and equipment changes may be associated with reduced mortality.

## Key Points

**Question:**

Are repetitive head impacts during a professional football career associated with mortality among National Football League players?

**Findings:**

In this cohort study of 13 912 National Football League players, a 25% increase in repetitive head impacts during a professional football career was associated with a statistically significant increase in the hazard ratio of death.

**Meaning:**

The findings suggest that repetitive head impacts are associated with an increase in the risk of all-cause mortality among professional football players.

## Teaser

This cohort study examines the association between repetitive head impacts from professional football play and all-cause mortality among National Football League players.

## Introduction

Sports are a major source of physical activity and entertainment for millions of individuals in the US. In general, participating in sports and other forms of exercise is seen as beneficial for physical, social, and psychological health.^1,2^ However, there are also risks associated with sports, particularly acute traumatic and repetitive injuries, yet the long-term adverse health outcomes associated with sports participation are understudied. Recently, national attention has focused on the short- and long-term effects of repetitive head impacts, including concussions, neurodegenerative disease, and chronic traumatic encephalopathy. Head impacts and injuries can occur in any sport but are prevalent among US-style football players. In 2018, an estimated 5.22 million persons in the US of all ages participated in tackle football according to the Outdoor Foundation.^3^ At the high school level, participation in football resulted in an estimated 55 007 concussions during the 2005 season.^4^ Although exposure to subconcussive blows is not known, there is evidence that these types of blows pervade the game of US-style football and that their cumulative neurodegenerative toll is also substantial.^5,6^ Several clinical studies^5,6,7^ of high school football players have shown biochemical and neurological changes as well as blood-barrier disruption after subconcussive hits to the head. It is important to understand the risks associated with repetitive head injuries to improve the long-term health outcomes in children, adolescents, and adults involved in contact and collision sports. Robust data on youth athletes do not exist; however, detailed data are available on US-style football players in the National Football League (NFL).

Elite athletes tend to have a lower overall mortality rate than the general population, which is often attributed to increased routine physical activity.^8,9^ Among elite US-style football players, there is a large body of literature^10,11^ surrounding risk factors for immediate injury during play or practice. However, relatively few studies^12,13,14^ have examined the health status of NFL players during the years after careers have ended. This is particularly concerning because many of the health outcomes associated with head impacts take many years to develop and may not be clinically apparent for several decades. Several studies^15,16,17^ involving small samples of NFL players have found associations between concussions or chronic traumatic encephalopathy and depression, suicide, and dementia. However, these long-term health outcomes are difficult to study systematically because of the lag time from the injury to the onset of symptoms and the relatively small number of cases. In addition, selection bias and recall bias are major limitations of these studies and make it difficult to generalize the conclusions.

Repetitive head impacts, not just concussions, have also been shown to be associated with adverse health outcomes.^5,6,7,18^ However, repetitive head impacts are difficult to measure. Researchers from Boston University developed the cumulative head impact index (CHII), which combines self-reported football exposure, player positions, levels of play, and helmet accelerometer studies.^19^ The CHII has been validated as a predictor of later-life neurobehavioral and cognitive impairment in NFL players.^20,21^ A study^22^ has shown that the number of NFL seasons played, a rough proxy for professional football head impacts, was associated with age at death in a U-shaped pattern. More seasons played was associated with a younger age at death up to the tenth season; then, the association switched direction, indicative of a survivorship bias among NFL players. However, the analysis only included deceased NFL players and did not capture the variation in head impacts experienced by players with more game time and across the positions.

We present an analysis of the association between repetitive head impacts of professional football players during an NFL career and mortality in more than 13 000 current and former NFL players during the 1969 to 2017 football seasons. The results of this study highlight on-field risk factors for mortality among US-style football players. This information, combined with that of other observational and experimental studies, could lead to the development of new technologies, protective equipment, and policies to make football and all sports safer to play at all ages and levels.

## Methods

### Data Collection

For this cohort study, data were collected from Pro Football Reference,^23^ an open-access online database maintained by Sports Reference LLC that includes playing statistics from the more than 23 000 past and present NFL players, with meticulously recorded data starting in 1922. After obtaining permission from the company to systematically acquire data from the website using a computer program, we custom-built a web crawler to retrieve and compile this data set. Variables included in this analysis were date of birth, date of death, player position, body mass index (BMI) (calculated as weight in kilograms divided by height in meters squared), and height. The study was initiated with data collection started on July 13, 2017, and follow-up ended on July 1, 2018. Because data were collected from a publicly available website, it was determined by the Syracuse University institutional review board, Syracuse, New York, that review, oversight, and informed consent were not required. The study followed the Strengthening the Reporting of Observational Studies in Epidemiology (STROBE) reporting guideline.

### Time at Risk Determination

Time spent in padded practice and field time were calculated based on information provided in the NFL Players Association Collective Bargaining Agreement, which has been updated approximately every 12 years beginning in 1968 (Table 1).^24,25,26,27,28,29,30^ The main analysis only included players in seasons 1986 to 2017, in which padded practice time could be accurately calculated from the collective bargaining agreement. A secondary analysis included players in all seasons, with the padded practice time from the first collective bargaining agreement applied to the earlier seasons.

**Table 1.** Padded Practice Time Allowed in National Football League Players Association CBAs^a^

CBA edition	Season years	Season, h/season		Postseason practice, h/wk	Games, h/game	Degree of regulation
		Off	Regular practice			
2011-2020^24^	2012 to Present	20	20	0.75	1	High
2002-2008^25^	2003-2011	24	23	2.00	1	High
1993-2005^26^	1999-2002	24	23	2.00	1	High
1993-2005^26^	1994-1998	24	23	4.00	1	Moderate
1982-1987^27^	1983-1993	29	23	4.00	1	Moderate
1974-1982,^29^ 1970-1974^28^	1972-1982	30	25	5.00	1	Low
1968-1970^30^^,^^a^	1969-1971	30	25	5.00	1	Low

Abbreviation: CBA, collective bargaining agreement.

^a^ Before 1968, time at risk from the 1968 CBA was applied.

### Professional Football CHII Calculation

A score was used to quantify the amount and severity of repeated head impacts based on the CHII (eTable 1 in the Supplement).^19^ The CHII combined reported football history with helmet accelerometer studies.^19^ We developed a professional football CHII (pfCHII) based on the CHII method specifically to quantify the relative amount and severity of head impacts accumulated during a professional football career. A pfCHII was calculated for each player as outlined in the equation below. eTable 2 in the Supplement compares the calculation of the CHII and pfCHII.
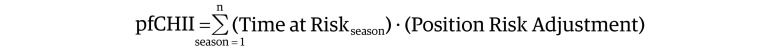
No self-reported data were used; the numbers of seasons and games played were determined from Pro Football Reference records. Time at risk was calculated by summing the exposure hours for each player using the playing time outlined in Table 1. The position risk adjustment was developed from helmet accelerometer studies to quantify the relative frequency and severity of repetitive head impacts by position and was included as described previously.^19^ Only professional football exposures were used for the pfCHII calculation.

### Statistical Analysis

Demographic characteristics were compared across position categories using a χ^2^ test for categorical variables, an analysis of variance for normally distributed continuous variables, and a Kruskal-Wallis test for nonnormally distributed continuous variables. Cox proportional hazards regression was used to calculate the hazard ratios (HRs) of death for an increasing pfCHII both unadjusted and adjusted for year of birth, BMI, and height. The time scale started when participants reached the age of 20 years. Because of the skewed distribution, the pfCHII was log transformed before inclusion in the regression. The squared term (log[pfCHII])^2^ was also included because previous research has shown the presence of a healthy worker effect among NFL players with longer careers.^22,31^ The healthy worker effect in football results from 3 factors: selection into the profession, physical benefits from the profession, and healthier players who are able to stay in the profession.^32^ The variables included in the model were chosen a priori based on previous studies.^22,33^ Players with missing data were eliminated from the regression analysis (Figure). A secondary analysis was performed including all players with complete data in the Pro Football Reference (1922-2017 seasons). Two sensitivity analyses were completed: one used a Cox proportional hazards regression model with position-fixed effects, and the other used a Cox proportional hazards regression model with birth cohort–fixed effects (instead of continuous birth year). Statistical analysis was performed from January to June 2019. All tests were 2-sided, and *P* ≤ .05 was considered statistically significant. All analyses were completed in Stata, version 14 (StataCorp LLC).^34^

**Figure.**
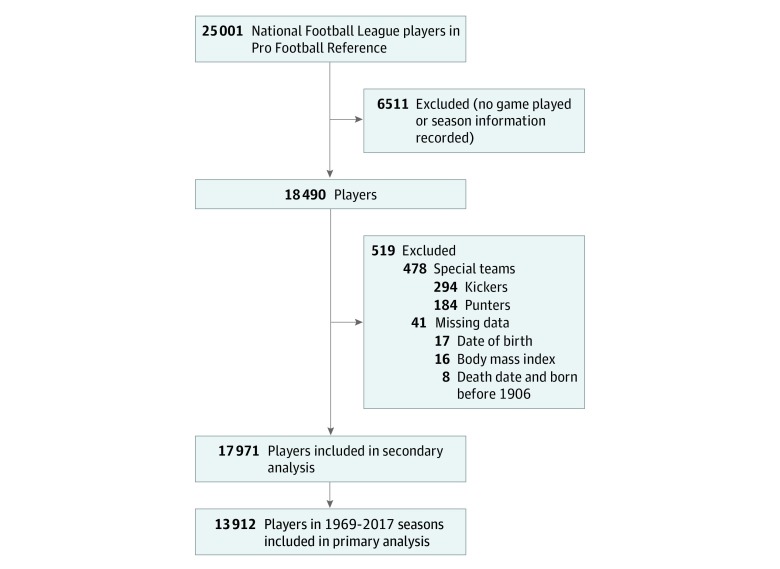
Flowchart of National Football League Players in the Pro Football Reference for the 1922 to 2017 Seasons

## Results

There were 25 001 players listed in the Pro Football Reference database, of whom 6511 (26.0%) were on a team roster but did not complete a full season. Of the 18 490 remaining players, 519 (2.8%) were missing data needed for the regression analysis. All NFL players were male; to date, there have been no female NFL players. As of 2018, the oldest living man in the world was born in 1906; therefore, the 8 players without a death date born before 1906 were eliminated from the analysis. There were no helmet accelerometer data on players from special teams (kickers and punters); thus, a pfCHII could not be calculated. The remaining 17 971 players were included in the secondary analysis (eTable 3 in the Supplement), and the 13 912 players (77.4%) in the 1969-2017 seasons were included in the main analysis (Figure).

Demographic characteristics except for the pfCHII were calculated for 14 366 players with complete follow-up. The pfCHII was calculated for 13 912 players (eliminating the 454 specials teams players). The mean (SD) age of players who participated in the 1969 to 2017 seasons was 47.3 (14.8) years, and 763 of 14 366 players (5.3%) had died as of July 1, 2018. The distribution of the age of death by position is shown in eFigure 1 in the Supplement. The mean (SD) BMI was 29.6 (3.9) (Table 2). The median pfCHII was 32.63 (interquartile range [IQR], 13.71-66.12) and ranged from 14.18 for wide receivers to 84.34 for offensive linemen (Table 2 and eFigure 2 in the Supplement). When including all seasons, the median pfCHII was 30.71 (IQR, 13.51-3.22), ranging from 12.85 for quarterbacks to 69.01 for offensive linemen (eTable 3 in the Supplement). All demographic characteristics were significantly different across the positions.

**Table 2.** National Football League Player Demographic Characteristics During the 1969 to 2017 Seasons

Characteristic^a^	Total (N = 14 366)	Quarterback (n = 662)	Wide receivers (n = 2946)	Offensive backs or running backs (n = 1814)	Defensive backs (n = 2753)	Linebackers (n = 2093)	Linemen		Special teams (n = 454)
							Offensive (n = 1543)	Defensive (n = 2101)	
Position risk adjustment	NA	0.0579	0.0666	0.1159	0.1173	0.1926	0.2047	0.2449	NA
Seasons, median (IQR)	4 (2-7)	5 (2-9)	4 (2-7)	3 (2-6)	4 (2-7)	5 (2-7)	7 (4-9)	5 (2-8)	3 (1-7)
pfCHII, median (IQR)	32.63 (13.71-66.12)^b^	15.31 (6.25-32.02)	14.18 (7.19-27.77)	22.72 (11.71-44.77)	29.44 (13.84-52.84)	53.54 (24.65-91.87)	84.34 (53.84-116.50)	65.88 (31.35-119.50)	NA
Age as of July 1, 2018, mean (SD), y	47.3 (14.8)	50.9 (14.8)	47.0 (14.4)	49.1 (14.6)	45.5 (14.5)	47.2 (14.8)	49.8 (15.1)	44.8 (14.5)	50.8 (14.5)
Dead, No. (%)	763 (5.3)	32 (4.8)	107 (3.6)	116 (6.4)	107 (3.9)	102 (4.9)	120 (7.8)	154 (7.3)	25 (5.5)
Age at death, mean (SD), y	53.3 (14.6)	61.9 (12.6)	54.3 (14.7)	52.3 (13.4)	51.2 (15.4)	50.7 (16.1)	56.6 (13.3)	51.4 (14.5)	57.9 (10.5)
Body mass index, mean (SD)^c^	29.6 (3.9)	26.7 (1.5)	27.2 (2.5)	29.4 (2.2)	26.5 (1.5)	30.3 (1.6)	34.7 (3.2)	34.2 (3.4)	26.4 (1.9)
Height, mean (SD), cm	186.8 (6.4)	189.5 (4.1)	186.5 (6.7)	181.5 (4.7)	182.0 (4.1)	187.8 (3.7)	193.5 (4.3)	192.2 (4.1)	183.5 (5.6)

Abbreviations: IQR, interquartile range; NA, not applicable; pfCHII, professional football cumulative head impact index.

^a^ Seasons and pfCHII compared using the Kruskall-Wallis test; percentage dead compared using a χ^2^ test; and age, age at death, body mass index, and height compared using analysis of variance. All characteristics were statistically significantly different across the positions (*P* < .001).

^b^ N = 13 912.

^c^ Calculated as weight in kilograms divided by height in meters squared.

In the unadjusted Cox proportional hazards regression model for the seasons 1969 to 2017, a 1-log increase in pfCHII was associated with an 80% increase in the hazard of death (HR, 1.80; 95% CI, 1.09-2.97; *P* = .02). When expanding the analysis to all players, the unadjusted HR was 1.50 (95% CI, 1.21-1.86) (eTable 4 in the Supplement). After adjusting for year of birth, BMI, and height (all known risk factors for death), a 1-log increase in pfCHII was associated with an increase in the hazard of death (2.02; 95% CI, 1.21-3.37; *P* = .01) for seasons 1969 to 2017. An increasing BMI and earlier year at birth were associated with an increased hazard of death in both models (eTable 5 in the Supplement). The complete adjusted models are shown in eTable 5 in the Supplement. The pfCHII was weighted based on player position, with offensive and defensive linemen weighted heavily in the calculation. These players are at higher risk of mortality because of factors not associated with head impacts.^33^ The sensitivity analysis added player-fixed effects to the model, which did not substantially change the association between the pfCHII and mortality (eTable 6 in the Supplement). We also examined birth year as a categorical variable instead of a continuous variable; this also did not substantially change the association between the pfCHII and mortality (eTable 6 in the Supplement).

The HR for log of pfCHII^2^ was slightly less than 1 in each model, indicating a nonlinear association between the log of pfCHII and the risk of death. The HR for log of pfCHII^2^ was statistically significant in all models (0.91 [95% CI, 0.85-0.98], *P* = .01 for the 1969-2017 adjusted model; 0.94 [95% CI, 0.91-0.97], *P* < .001 for the 1922-2017 unadjusted model; 0.96 [95% CI, 0.93-0.99], *P* = .02 for the adjusted 1922-2017 model) except the unadjusted model for seasons 1969 to 2017 (0.95; 95% CI, 0.88-1.01; *P* = .12) (eTable 4 in the Supplement). The chief purpose of this quadratic term was to control for the confounding risk associated with a healthy (NFL) worker effect on hazard of death observed in the literature.^22,31,32^ With this term, we obtained an estimate for the HR of the variable of interest, log of pfCHII, that was conditional on the healthy worker effect and, therefore, not survivorship biased.^22^ Estimated concavity from the quadratic term was slight and mitigated only a low proportion of the reported increase in hazard associated with increases in the linear term pfCHII (eTable 7 in the Supplement).

## Discussion

This analysis included 13 912 NFL players who participated in the 1969 to 2017 football seasons. The results showed that an index value of repetitive head impacts during a professional football career was associated with a statistically significant and substantially increased risk for mortality. The association persisted even when allowing for the healthy worker effect to provide a conservative estimate of the association of head impacts with death. For the 1969 to 2017 football seasons, a 1% increase in pfCHII was associated with a 0.7% (95% CI, 0.2%-1.2%) increase in the risk of death, a 25% increase in pfCHII was associated with a 16% (95% CI, 4%-30%) increase in the risk of death, and a 100% increase in pfCHII was associated with a 56% (95% CI, 13%-114%) increase in the risk of death (eTable 7 in the Supplement). To increase the pfCHII by 1%, a player with 1 season of practices would need to play only 1 additional game the next season. To increase the pfCHII by 25%, a 1-season player would need to add only half of a regular season of game play and practice time the next season. Consistent with previous studies, increasing BMI was statistically significantly associated with increased hazard of death in all models.^9,33,35,36^

This study minimized the selection and recall biases found in previous studies among NFL players. Several studies^33,37^ examined mortality among NFL players using the NFL pension fund, which only included players with at least 5 seasons of play in the NFL. Other studies^13,19,24,38,39^ focusing on chronic traumatic encephalopathy and other neurodegenerative conditions relied heavily on recall of players or family members regarding positions and seasons played; these studies were often conducted after neurodegenerative symptoms had started to occur. Autopsy studies rely on self-selection or family selection such that families suspecting the presence of a neurodegenerative condition may be more likely to select into the autopsy study. Castellani et al^38^ referred to self-selection bias as axiomatic to brain autopsy studies. Use of the collective bargaining agreements and Pro Football Reference eliminated these biases.

Future directions for this research include further validation of the pfCHII using film-based correlation and clinical studies. The pfCHII could also be used to identify practices and on-field playing characteristics that put certain players or positions at a high risk of increased exposure to repetitive head impacts. Further analysis examining the association between repetitive head impacts and cause of death is needed. It is also important to consider total head impacts because of football participation during a lifetime rather than just those associated with a professional football career. In addition, examination of the association between repetitive head impacts and mortality risk, specifically neurodegenerative mortality, by helmet use, tackling, and other policies aimed at reducing head impacts is needed.

The results of this study suggest that efforts by policy makers to directly reduce repetitive head impacts may be beneficial to football player health.^40^ Policies consistent with this objective are being piloted in the National Collegiate Athletic Association and NFL but should also be implemented for high school and youth leagues.^41,42^ In 2016, for example, the Ivy League banned full-contact tackling from all regular season football practices. In 2018, the NFL instituted a rule that penalized players who initiate contact by lowering their head. The NFL also met with the National Collegiate Athletic Association in that year to align football player safety rules across the 2 levels. Substantial questions remain regarding whether more fundamental changes should be considered and whether these considerations should be imposed by leagues or by federal legislation and regulation. Traditionally, federal agencies such as the Occupational Safety and Health Administration have allowed leagues to self-regulate the health and safety of players, with exceptional cases being governed largely by tort law. Given recent research findings regarding public health issues raised by contact sport participation, this may not be an optimal approach within the modern landscape.

### Limitations

This study has limitations. Repetitive head impacts in this study were imperfectly measured, especially for the seasons before 1969, which were not well regulated. It was unclear how well the pfCHII reflected actual player head impact experiences. Future analysis could use video recordings to estimate how well the pfCHII reflects player experiences. However, the CHII method has been validated as a marker of head impact–associated health outcomes in this population.^20,21^ We did not calculate the pfCHII as a time-varying covariate, but most NFL players retired when they reached an age in the mid-twenties. In addition, the pfCHII does not incorporate college, high school, or youth exposures to head impacts. It may be important to consider total head impacts from football participation during a lifetime instead of just those associated with a professional football career.^13,43^ In contrast, there is some evidence that high school level participation in football may not be associated with neurodegenerative risk.^44,45^

Race/ethnicity was not recorded in Pro Football Reference; thus, it could not be adjusted in our analysis. However, African American football players were integrated into NFL precursor leagues beginning in 1908. Documentation from the NFL pension fund used in an analysis by Baron et al^33^ showed that the racial/ethnic breakdown between the years 1960 and 2007 was approximately 60% white players and 39% African American players. Another analysis^46^ performed outside of the league and its affiliates suggested that integration was representatively insignificant until 1960, increasing from 12% African American players in 1960 to 67% African American players in 1997. In the general US population, African American males have a lower life expectancy than white males.^47^ However, the association between race/ethnicity and mortality from repetitive head impacts has not yet been examined with sufficient sample size.

There could also be inaccuracies in the crowdsourced data from Pro Football Reference. Players with shorter careers, and by definition a lower pfCHII, were more likely to be listed in the database as alive when they were dead. The estimates found in this analysis could be slightly overestimated because of this information bias. Future analysis should examine the accuracy of mortality records in Pro Football Reference.

In addition, this analysis examined all-cause mortality not limited to neurodegenerative causes. By definition, the pfCHII is associated with length of exposure and position. These latter inputs may, in turn, be associated with other mortality risk factors such as hypertension and heart disease.^48^ Future analysis examining the change in BMI over time and risk of all-cause and cause-specific mortality is needed. The association between neurodegenerative causes and repetitive head impacts may be greater than the association found here.

## Conclusions

The findings suggest that an increase in repetitive head impacts is associated with an increased hazard of death among NFL players. Reduction in repetitive head impacts from playing football or other activities through additional rule and equipment changes may be associated with reduced mortality.

## Notes

Supplement. **eTable 1.** Summary Data of Head Impacts From Helmet Accelerometer Studies **eTable 2.** Comparison of CHII and pfCHII Calculations **eTable 3.** National Football League Player Demographics, 1922-2017 Seasons (n=18 449) **eTable 4.** Risk Factors for Mortality Among National Football League Players, 1922-2017 Seasons (n=17 971) **eTable 5.** Full Cox Proportional Hazards Regression Models for Mortality Among National Football League Players **eTable 6.** Sensitivity Analyses of the Cox Proportional Hazards Regression Models for Mortality Among National Football League Players **eTable 7.** Relative Change in Hazard of Death Calculation **eFigure 1.** Density Plot of Age of Death for NFL Players, 1922-2017 Seasons (n=3974) **eFigure 2.** Descriptive Kaplan-Meier Curve by pfCHII Quartile for NFL Players, 1968-2017 Seasons (n=13 912)
