# Incorrect Date in the Methods Section

**DOI:** 10.1001/jamanetworkopen.2020.10560

**Published:** 2020-06-02

In the Original Investigation, “Association of Professional Football Cumulative Head Impact Index Scores With All-Cause Mortality Among National Football League Players,”^1^ published on May 11, 2020, in the Time at Risk Determination subsection of the Methods section, the main analysis included players in seasons 1969 to 2017, not 1986 to 2017. This article has been corrected.
