# Supplementary material for: Association of Professional Football Cumulative Head Impact Index Scores With All-Cause Mortality Among National Football League Players
Source: JAMA Netw Open. 2020 May 11;3(5):e204442. doi: 10.1001/jamanetworkopen.2020.4442 (PMC7215260; doi:10.1001/jamanetworkopen.2020.4442)
Supplement: Supplement. — eTable 1. Summary Data of Head Impacts From Helmet Accelerometer Studies eTable 2. Comparison of CHII and pfCHII Calculations eTable 3. National Football League Player Demographics, 1922-2017 Seasons (n=18 449) eTable 4. Risk Factors for Mortality Among National Football League Players, 1922-2017 Seasons (n=17 971) eTable 5. Full Cox Proportional Hazards Regression Models for Mortality Among National Football League Players eTable 6. Sensitivity Analyses of the Cox Proportional Hazards Regression Models for Mortality Among National Football League Players eTable 7. Relative Change in Hazard of Death Calculation eFigure 1. Density Plot of Age of Death for NFL Players, 1922-2017 Seasons (n=3974) eFigure 2. Descriptive Kaplan-Meier Curve by pfCHII Quartile for NFL Players, 1968-2017 Seasons (n=13 912) [file jamanetwopen-3-e204442-s001.pdf]

Supplementary Online Content

Kmush BL, Mackowski M, Ehrlich J, Walia B, Owora A, Sanders S. Association of professional football cumulative head impact index scores with all-cause mortality among National Football League players. *JAMA Netw Open*. 2020;3(5):e204442. doi:10.1001/jamanetworkopen.2020.4442

eTable 1. Summary Data of Head Impacts From Helmet Accelerometer Studies

eTable 2. Comparison of CHII and pfCHII Calculations

eTable 3. National Football League Player Demographics, 1922-2017 Seasons (n=18 449)

eTable 4. Risk Factors for Mortality Among National Football League Players, 1922-2017 Seasons (n=17 971)

eTable 5. Full Cox Proportional Hazards Regression Models for Mortality Among National Football League Players

eTable 6. Sensitivity Analyses of the Cox Proportional Hazards Regression Models for Mortality Among National Football League Players

eTable 7. Relative Change in Hazard of Death Calculation

eFigure 1. Density Plot of Age of Death for NFL Players, 1922-2017 Seasons (n=3974)

eFigure 2. Descriptive Kaplan Meier Curve by pfCHII Quartile for NFL Players, 1968-2017 Seasons (n=13 912)

This supplementary material has been provided by the authors to give readers additional information about their work.

© 2020 Kmush BL et al. *JAMA Network Open.*

eTable 1. Summary Data of Head Impacts From Helmet Accelerometer Studies

**Position**	**Weighted Mean Impacts Per Season**[a]	**Position Risk Adjustment**[b]
Quarterbacks	206	0.0579
Wide Receivers	237	0.0666
Offensive Backs or Running Backs	412	0.1159
Defensive Backs	417	0.1173
Linebackers	685	0.1926
Offensive Linemen	728	0.2047
Defensive Linemen	871	0.2449
Special Teams	NOT INCLUDED	NOT INCLUDED

[a]As reported in Montinegro 2017.

[b]Position proportion of the total mean impacts per season.

© 2020 Kmush BL et al. *JAMA Network Open.*

eTable 2. Comparison of CHII and pfCHII Calculations

CHII[a,b]	$\sum_{\text{youth position}=1}^{3} \text{Time at Risk} \times \text{Weighted Mean Impacts per Season}_{\text{position}}$ $+$ $\sum_{\text{high school position}=1}^{3} \text{Time at Risk} \times \text{Weighted Mean Impacts per Season}_{\text{position}}$ $+$ $\sum_{\text{college position}=1}^{3} \text{Time at Risk} \times \text{Weighted Mean Impacts per Season}_{\text{position}}$ $+$ $\sum_{\text{professional position}=1}^{3} \text{Time at Risk} \times \text{Weighted Mean Impacts per Season}_{\text{position}}$
pfCHII[c,d]	$\sum_{\text{professional season}=1}^{n} \text{Time at Risk}_{\text{season}} \times \text{Position Risk Adjustment}$

[a]Time at Risk in CHII=% games played at position $\times$ Total no. of seasons

[b]Position's Weighted Mean Impacts per Season is reported in Supplementary Table 1 for College and Professional Positions, and in Montinegro et. Al. 2017 for Youth and High School Positions

[c]Time at Risk in pfCHII=Hours at Risk as reported in Table 1 of the main manuscript

[d]Position Risk Adjustment reported in Supplementary Table 1, derived from the Position's Weighted Mean Impacts per season.

CHII-cumulative head impact index; pfCHII-professional football cumulative head impact index

© 2020 Kmush BL et al. *JAMA Network Open.*

eTable 3. National Football League Player Demographics, 1922-2017 Seasons (n=18 449)

Characteristic[a]	Total (n=18,449)	Quarterback (n=896)	Wide Receivers (n=3,104)	Offensive Backs or Running Backs (n=2,914)	Defensive Backs (n=3,367)	Linebackers (n=2,496)	Offensive Linemen (n=2,388)	Defensive Linemen (n=2,806)	Special Teams (n=478)
Position Risk Adjustment	NA	0.0579	0.0666	0.1159	0.1173	0.1926	0.2047	0.2449	NA
Seasons in NFL, median (IQR)	4 (2-7)	4 (2-8)	4 (2-7)	3 (1-5)	4 (2-7)	4 (2-7)	5 (3-8)	4 (2-7)	3 (1-7)
pfCHII, median (IQR)	30.71 (13.51-63.22)[b]	12.85 (5.59-27.53)	14.09 (7.15-27.67)	19.70 (7.77-39.06)	26.86 (13.49-48.44)	51.62 (24.56-89.75)	69.01 (39.30-106.4)	58.04 (28.41-111.4)	NA
Age, mean (SD), y as of 7/1/2018	49.4 (16.5)	53.7 (16.8)	48.0 (15.2)	52.9 (17.5)	47.3 (16.1)	49.0 (16.4)	52.5 (17.2)	46.8 (16.7)	51.7 (15.1)
Dead, n (%)	3,974 (21.5%)	203 (22.7%)	169 (5.4%)	1,000 (34.3%)	579 (17.2%)	398 (15.9%)	836 (35.0%)	756 (26.9%)	33 (6.9%)
Age, mean (SD), y at death	68.6 (16.0)	72.2 (14.0)	59.6 (15.5)	70.0 (15.7)	68.1 (16.2)	67.3 (17.3)	69.8 (14.7)	67.7 (17.0)	60.9 (11.3)
Body Mass Index, mean (SD), kg/m^2	29.0 (3.8)	26.4 (1.5)	27.1 (2.5)	28.3 (2.5)	26.3 (1.5)	30.0 (1.7)	32.9 (3.8)	32.3 (4.5)	26.5 (1.9)
Height, mean (SD), cm	186.2 (6.3)	187.9 (5.1)	186.6 (6.7)	181.5 (4.6)	181.9 (4.3)	187.4 (3.8)	191.0 (5.7)	190.8 (4.9)	183.6 (5.6)

[a]Seasons and pfCHII compared using the Kruskall-Wallis test; percent dead compared using a χ^2 test; age, age at death, body mass index, and height compared using ANOVA. All characteristics were statistically significantly different across the positions (p<0.001).

[b]N=17,971

pfCHII-professional football cumulative head impact index; IQR-Interquartile range; NA-not applicable.

© 2020 Kmush BL et al. *JAMA Network Open.*

eTable 4. Risk Factors for Mortality Among National Football League Players, 1922-2017 Seasons (n=17 971)

Characteristic	Unadjusted Cox Proportional Hazards Regression Model[a]			Adjusted Cox Proportional Hazards Regression Model[b]		
	HR	95% CI	p-value	HR	95% CI	p-value
Log pfCHII	1.50	1.21-1.84	<0.001	1.24	1.00-1.55	0.05
$(\text{Log pfCHII})^2$	0.94	0.91-0.97	<0.001	0.96	0.93-0.99	0.02

[a]Log pfCHII and $(\text{log pfCHII})^2$ were included in the unadjusted model.

[b]Adjusted model included log pfCHII, $(\text{log pfCHII})^2$, year of birth, BMI, and height.

pfCHII-professional football cumulative head impact index; HR-hazard ratio; CI-confidence interval; BMI-body mass index.

© 2020 Kmush BL et al. *JAMA Network Open.*

eTable 5. Full Cox Proportional Hazards Regression Models for Mortality Among National Football League Players

Characteristic	1969-2017 seasons (N=13,912)				1922-2017 season (N=17,971)		
	HR	95% CI	p-value		HR	95% CI	p-value
Log pfCHII	2.02	1.21-3.37	0.007		1.24	1.00-1.55	0.05
(Log pfCHII)^2	0.91	0.85-0.98	0.009		0.96	0.93-0.99	0.02
Birth Year	0.99	0.98-1.00	0.004		0.98	0.97-0.98	<0.001
BMI (kg/m^2)	1.09	1.07-1.12	<0.001		1.07	1.06-1.09	<0.001
Height (cm)	1.00	0.99-1.01	0.87		1.00	1.00-1.01	0.29

[a]Log pfCHII and (log pfCHII)^2 were included in the same unadjusted model.

[b]Adjusted model included log pfCHII, (log pfCHII)^2, year of birth, BMI, and height.

pfCHII-professional football cumulative head impact index; HR-hazard ratio; CI-confidence interval; BMI-body mass index.

© 2020 Kmush BL et al. *JAMA Network Open.*

eTable 6. Sensitivity Analyses of the Cox Proportional Hazards Regression Models for Mortality Among National Football League Players

Characteristic	1969-2017 seasons (N=13,912)							1922-2017 season (N=17,971)					
	HR	95% CI	p-value	HR	95% CI	p-value		HR	95% CI	p-value	HR	95% CI	p-value
Log pfCHII	1.96	1.16-3.31	0.01	2.01	0.20-3.36	0.008		1.27	1.01-1.59	0.04	1.21	0.97-1.50	0.09
(Log pfCHII)^2	0.91	0.85-0.98	0.02	0.91	0.85-0.98	0.009		0.96	0.93-0.99	0.02	0.97	0.94-1.00	0.04
Birth Year	0.99	0.98-0.99	0.003	-	-	-		0,98	0.97-0.98	<0.001	-	-	-
BMI (kg/m^2)	1.10	1.06-1.14	<0.001	1.09	1.06-1.12	<0.001		1.08	1.06-1.10	<0.001	1.07	1.05-1.08	<0.001
Height (cm)	1.01	0.99-1.02	0.39	1.00	0.99-1.02	0.86		1.01	1.00-1.01	0.15	1.00	1.00-1.01	0.29
Position Category													
Quarterback	Ref.	-	-	-	-	-		Ref.	-	-	-	-	-
Wide Receiver	1.15	0.77-1.72	0.50	-	-	-		0.88	0.71-1.08	0.22	-	-	-
Offensive Back/ Running Back	1.48	0.98-2.25	0.06	-	-	-		1.10	0.94-1.29	0.23	-	-	-
Defensive Back	1.43	0.94-2.18	0.10	-	-	-		1.10	0.93-1.30	0.28	-	-	-
Linebacker	1.07	0.69-1.65	0.77	-	-	-		0.94	0.78-1.13	0.48	-	-	-
Offensive Lineman	1.02	0.63-1.64	0.95	-	-	-		0.96	0.80-1.16	0.69	-	-	-
Defensive Lineman	1.46	0.91-2.35	0.11	-	-	-		1.12	0.95-1.34	0.19	-	-	-
Birth Cohort													
Prior to 1890	-	-	-	-	-	-		-	-	-	Ref.	-	-
1890-1899	-	-	-	-	-	-		-	-	-	0.43	0.19-0.97	0.04
1900-1909	-	-	-	-	-	-		-	-	-	0.42	0.19-0.94	0.03
1910-1919	-	-	-	-	-	-		-	-	-	0.31	0.14-0.70	0.005
1920-1929	-	-	-	Ref.	-	-		-	-	-	0.27	0.12-0.60	0.001
1930-1939	-	-	-	0.50	0.12-2.07	0.34		-	-	-	0.20	0.09-0.46	<0.001
1940-1949	-	-	-	0.40	0.10-1.65	0.21		-	-	-	0.13	0.06 0.29	<0.001
1950-1959	-	-	-	0.33	0.08 1.38	0.13		-	-	-	0.11	0.05-0.24	<0.001
1960-1969	-	-	-	0.29	0.07-1.21	0.09		-	-	-	0.10	0.04-0.23	<0.001
1970-1979	-	-	-	0.23	0.05-0.99	0.05		-	-	-	0.09	0.04-0.22	<0.001
1980-1989	-	-	-	0.35	0.08-1.53	0.16		-	-	-	0.13	0.05-0.33	<0.001
1990-1999	-	-	-	0.33	0.05-2.10	0.24		-	-	-	0.12	0.03-0.51	0.004

[a]Log pfCHII and (log pfCHII)^2 were included in the same unadjusted model.

[b]Adjusted model included log pfCHII, (log pfCHII)^2, year of birth, BMI, and height.

pfCHII-professional football cumulative head impact index; HR-hazard ratio; CI-confidence interval; BMI-body mass index.

© 2020 Kmush BL et al. *JAMA Network Open.*

eTable 7. Relative Change in Hazard of Death Calculation[a]

**(A)** **Increase in pfCHII**	**(B)** $A \times log(2.02)$	**(C)** $A^2 \times log(0.91)$	**(D)** $B + C$	**(E)** e^D	**Relative Change in Hazard of Death (95% CI)[b]**
1%	0.0070	0.0000	0.0070	1.007	+0.7 (0.2-1.2)%
25%	0.1572	-0.0048	0.1524	1.16	+16 (4-30)%
50%	0.2857	-0.0157	0.2699	1.31	+31 (8-60)%
75%	0.3943	-0.0299	0.3643	1.44	+44 (11-87)%
100%	0.4883	-0.0459	0.4424	1.56	+56 (13-114)%
2.718 unit[b]	0.7045	-0.0956	0.6089	1.84	+84 (18-185)%

[a]Calculations from the adjusted Cox model in Table 4, holding birth year, body mass index, and height constant.

[b]95% CI calculated in same manner as point estimate. Calculations not shown for clarity.

[c]A 1 unit increase in the log pfCHII corresponds to a 2.718 unit increase in pfCHII.

pfCHII-professional football cumulative head impact index; CI-Confidence Interval

© 2020 Kmush BL et al. *JAMA Network Open.*

eFigure 1. Density Plot of Age of Death for NFL Players, 1922-2017 Seasons (n=3974)

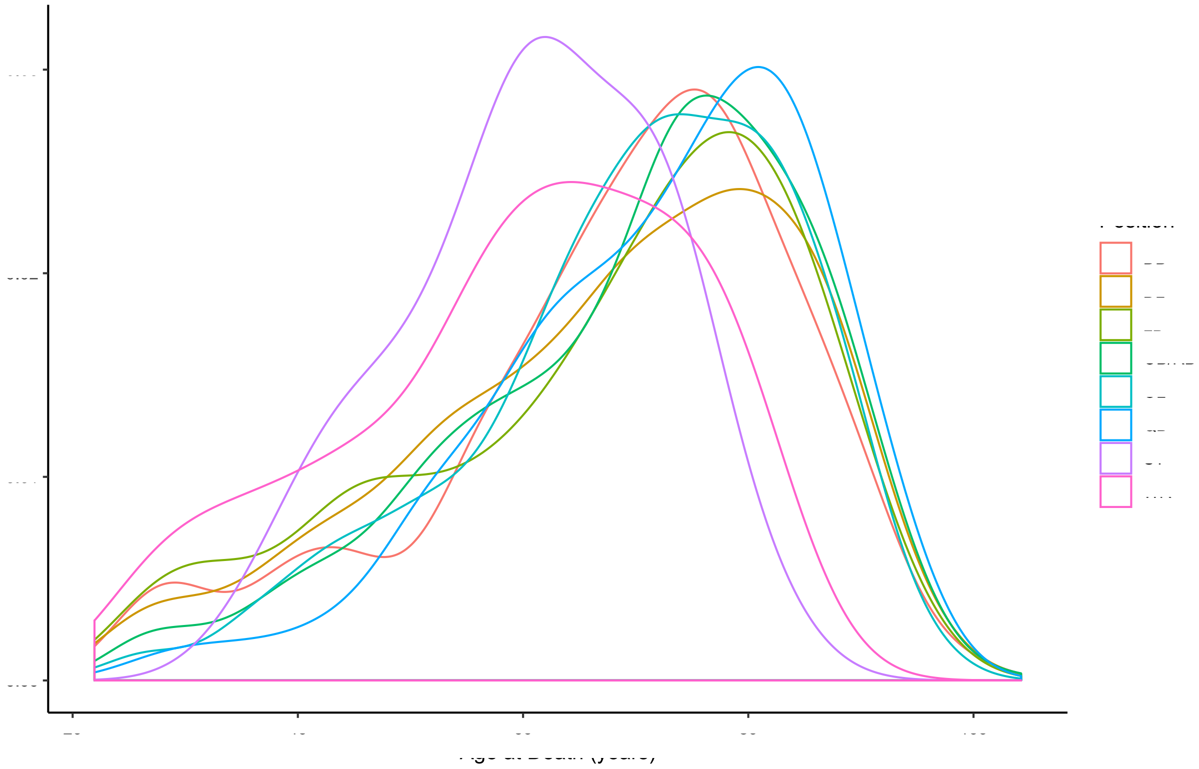

DB-Defensive Back (n=579)

DL-Defensive Linemen (n=756)

LB-Linebackers (n=398)

OB/RB-Offensive Backs or Running Backs (n=1,000)

OL-Offensive Linemen (n=836)

QB-Quarterbacks (n=203)

ST-Special Teams (n=33)

WR-Wide Receivers (n=169)

© 2020 Kmush BL et al. *JAMA Network Open.*

eFigure 2. Descriptive Kaplan Meier Curve by pfCHII Quartile for NFL Players, 1968-2017 seasons (n=13 912)

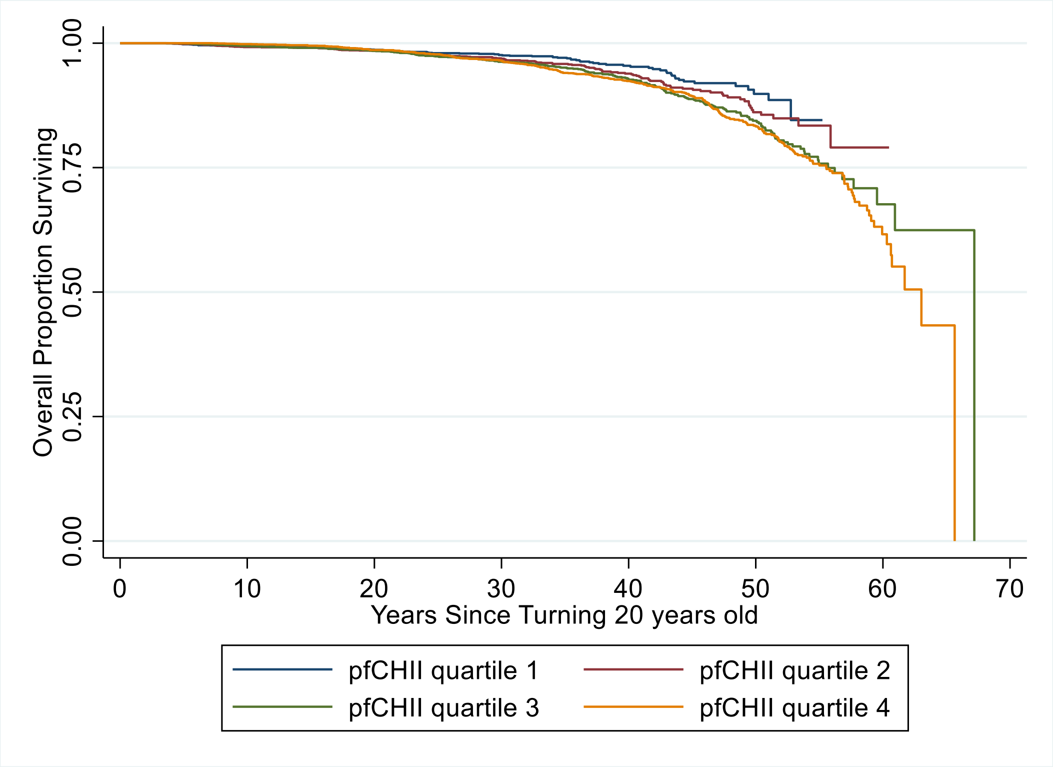

No. at Risk

**pfCHII Quartile**	**Years Since Turning 20 years old**						
	**0**	**10**	**20**	**30**	**40**	**50**	**60**
1	3,423	2589	1819	1247	472	106	1
2	3,206	2585	1836	1217	634	182	3
3	3,521	3092	2232	1561	892	319	19
4	3,762	3662	2876	2032	1247	516	40

pfCHII-professional football cumulative head impact index

© 2020 Kmush BL et al. *JAMA Network Open.*
